# Bringing to light the molecular evolution of *GUX* genes in plants

**DOI:** 10.1590/1678-4685-GMB-2018-0208

**Published:** 2020-03-23

**Authors:** Rafael Henrique Gallinari, Rafael Della Coletta, Pedro Araújo, Marcelo Menossi, Mariana Freitas Nery

**Affiliations:** 1 Universidade Estadual de Campinas, Instituto de Biologia, Departamento de Genética, Evolução, Microbiologia e Imunologia, Campinas, SP, Brazil.

**Keywords:** GUX, sugarcane, phylogeny, angiosperms, biofuels

## Abstract

Hemicellulose and cellulose are essential polysaccharides for plant development and major components of cell wall. They are also an important energy source for the production of ethanol from plant biomass, but their conversion to fermentable sugars is hindered by the complex structure of cell walls. The glucuronic acid substitution of xylan (GUX) enzymes attach glucuronic acid to xylan, a major component of hemicellulose, decreasing the efficiency of enzymes used for ethanol production. Since loss-of-function *gux* mutants of *Arabidopsis thaliana* enhance enzyme accessibility and cell wall digestion without adverse phenotypes, *GUX* genes are potential targets for genetically improving energy crops. However, comprehensive identification of GUX in important species and their evolutionary history are largely lacking. Here, we identified putative GUX proteins using hidden Markov model searches with the GT8 domain and a GUX-specific motif, and inferred the phylogenetic relationship of 18 species with Maximum likelihood and Bayesian approaches. Each species presented a variable number of GUX, and their evolution can be explained by a mixture of divergent, concerted and birth-and-death evolutionary models. This is the first broad insight into the evolution of *GUX* gene family in plants and will potentially guide genetic and functional studies in species used for biofuel production.

Plant evolution has been characterized by the development of complex organs and highly specialized cellular structures, including the complex plant cell wall (Sørensen *et al.*, 2010). This structure provides strength and support for the plant body, protects against pathogens and pests, regulates growth, minimizes water loss, and other mechanical and biochemical functions (Sarkar *et al.*, 2009). The cell wall, composed mainly by hemicellulose and cellulose, is very important to plant survival and accounts for most of their biomass (Park and Cosgrove, 2012; Loqué *et al.*, 2015). Consequently, from a technological perspective, the plant cell wall composed mainly of polysaccharides may serve as an important source of renewable energy. The problem is that its complexity decreases the efficiency of saccharification, *i.e.* the process of breaking down the polysaccharides into sugars that can be used as energy source (Jordan *et al.*, 2012; Yue *et al.*, 2014). For instance, the interaction between cellulose and xylan, one of the main components of hemicellulose, may impede the accessibility of enzymes that degrade cellulose to produce fermentable sugars (Simmons *et al.*, 2016).

At the molecular level, several genes that control the deposition and arrangement of the plant cell wall have been reported in *Arabidopsis thaliana*, such as the irregular xylem (IRX) genes *IRX8*, *IRX9*, *IRX14*, the genes fragile fiber 8/ irregular xylem 7 **(** *FRA8*), galacturonosyltransferase-like 1 (*PARVUS*) and glucuronic acid substitution of xylan (*GUX*) (Brown *et al.*, 2007; Lee *et al.*, 2007a b; Peña *et al.*, 2007; Mortimer *et al.*, 2010). While mutations in most of these genes only change the proportion of methylglucuronic acid (MeGlcA) and glucuronic acid (GlcA) attached to xylan, mutations on *GUX* genes were reported to reduce the presence of such residues that hinders the access of cellulases to biomass and to increase saccharification yield (Mortimer *et al.*, 2010; Lee *et al.*, 2012; Lyczakowski *et al.*, 2017). Importantly, these mutations did not interfere with plant development, making *GUX* genes potential targets for genetically engineering plant cell walls (Mortimer *et al.*, 2010; Lee *et al.*, 2012; Lyczakowski *et al.*, 2017). *GUX* genes comprise a multigene family, with five homologous genes annotated in the *Arabidopsis* genome (*AtGUX1-5*; Mortimer *et al.*, 2010; Rennie *et al.*, 2012), and at least one gene in the conifer *Picea glauca* (*PgGUX*; Lyczakowski *et al.*, 2017).

Accordingly, we performed *in silico* analyses to identify putative GUX proteins in different Angiosperm groups to infer their phylogenetic relationships to ultimately unravel their evolution from a molecular standpoint. Our results can guide future applied research with GUX in economically important biofuel crops, since the first step towards the production of genetically modified plants is to understand how widespread these genes are in a phylogenetic context, and also in how many copies they are present within the genome.

In order to reconstruct the phylogenetic relationship of the GUX family, we selected 16 angiosperm species (including six monocots and ten dicots) that are either model plants or important crops: thale cress (*Arabidopsis thaliana*), purple false brome (*Brachypodium distachyon*)*,* wild cabbage (*Brassica oleraceae*)*,* turnip (*Brassica rapa*)*,* sweet orange (*Citrus sinensis*)*,* flooded gum (*Eucalyptus grandis*)*,* soybean (*Glycine max*)*,* rice (*Oryza sativa*)*,* black cottonwood (*Populus trichocarpa*)*,* sugarcane (*Saccharum* spp.)*,* foxtail millet (*Setaria italica*)*,* potato (*Solanum tuberosum*)*,* sorghum (*Sorghum bicolor*)*,* cocoa (*Theobroma cacao*)*,* grape (*Vitis vinifera*) and maize (*Zea mays*). We also selected two bryophytes (the moss *Physcomitrella patens*, and the common liverwort *Marchantia polymorpha*) to serve as outgroups in the phylogenetics analysis. The accession numbers from each sequence are shown in Table 1.

**Table 1:** Number of GUX proteins found by HMMER analysis in each species, scientific name, accession number, clade that each protein belongs and the name that appears on the phylogenetic tree.

Scientific name (reference genome version)	# GUX	Accession number	Clade	Phylogenetic tree name
Arabidopsis thaliana* (TAIR10)	5	At3g18660	GUX 1	Arabidopsis_thaliana_GUX1
		At4g33330	GUX 2	Arabidopsis_thaliana_GUX2
		At1g54940.1	GUX 3	Arabidopsis_thaliana_GUX3
		At1g77130.1	GUX 4	Arabidopsis_thaliana_GUX4
		At1g08990.1	GUX 5	Arabidopsis_thaliana_GUX5
*Brachypodium distachyon†* (v3.1)	4	Bradi2g56810.1	GUX 1	Brachypodium_distachyon1
		Bradi1g72350.1	GUX 2	Brachypodium_distachyon2
		Bradi2g24737.4	GUX 3	Brachypodium_distachyon3
		Bradi3g45800.7	GUX X	Brachypodium_distachyonXA
		Bradi5g27680.1	GUX X	Brachypodium_distachyonXB
*Brassica oleraceae†* (v1.0)	7	Bol030957	GUX 1	Brassica_oleraceae1
		Bol013572	GUX 2	Brassica_oleraceae2A
		Bol017534	GUX 2	Brassica_oleraceae2B
		Bol009658	GUX 3	Brassica_oleraceae3
		Bol006577	GUX 4/5	Brassica_oleraceae5A
		Bol022153	GUX 4/5	Brassica_oleraceae5B
		Bol022154	GUX 4/5	Brassica_oleraceae5C
*Brassica rapa†* (v1.3)	10	Brara.E02330.1	GUX 1	Brassica_rapa1A
		Brara.A02917.1	GUX 1	Brassica_rapa1B
		Brara.A00465.1	GUX 2	Brassica_rapa2A
		Brara.H01273.1	GUX 2	Brassica_rapa2B
		Brara.F01545.1	GUX 3	Brassica_rapa3A
		Brara.H02280.1	GUX 3	Brassica_rapa3B
		Brara.B02173.1	GUX 3	Brassica_rapa3C
		Brara.I01695.1	GUX 4	Brassica_rapa4
		Brara.I05282.1	GUX 4/5	Brassica_rapa5A
		Brara.H02850.1	GUX 4/5	Brassica_rapa5C
*Citrus sinensis†* (v1.1)	3	orange1.1g006648m	GUX 1	Citrus_sinensis1
		orange1.1g007705m	GUX 2	Citrus_sinensis2
		orange1.1g043696m	GUX 3	Citrus_sinensis3
*Eucalyptus grandis†* (v2.0)	4	Eucgr.H04942.1	GUX 1	Eucalyptus_grandis1
		Eucgr.F00232.1	GUX 2	Eucalyptus_grandis2
		Eucgr.F02737.1	GUX 3	Eucalyptus_grandis3
		Eucgr.L01540.1	GUX 4	Eucalyptus_grandis4
*Glycine max†* (Wm82.a2.v1)	11	Glyma.04G214400.1	GUX 1	Glycine_max1A
		Glyma.06G151900	GUX 1	Glycine_max1B
		Glyma.05G060700.1	GUX 1	Glycine_max1C
		Glyma.05G190200.1	GUX 1	Glycine_max1D
		Glyma.17G242500.1	GUX 2	Glycine_max2A
		Glyma.14G082500.1	GUX 2	Glycine_max2B
		Glyma.04G038500.1	GUX 2	Glycine_max2C
		Glyma.02G238200.1	GUX 3	Glycine_max3A
		Glyma.14G122600.1	GUX 3	Glycine_max3B
		Glyma.19G235600.1	GUX 4	Glycine_max4A
		Glyma.10G154600.1	GUX 4	Glycine_max4B
*Marchantia polymorpha†* (v3.1)	1	Mapoly0120s0025.1	OUTGROUP	Marchantia_polymorpha_OUTGROUP
*Oryza sativa†* (v7_JGI)	3	LOC_Os01g65780.2	GUX 1	Oryza_sativa1
		LOC_Os03g08600.1	GUX 2	Oryza_sativa2
		LOC_Os02g35020.1	GUX X	Oryza_sativaX
*Physcomitrella patens†* (v3.3)	1	Pp3c1_28970V3.1	OUTGROUP	Physcomitrella_patens
*Populus trichocarpa†* (v3.1)	6	Potri.007G107200.1	GUX 1	Populus_trichocarpa1A
		Potri.005G061600.5	GUX 1	Populus_trichocarpa1B
		Potri.014G029900.1	GUX 2	Populus_trichocarpa2
		Potri.005G187900.1	GUX 3	Populus_trichocarpa3
		Potri.005G033500.1	GUX 4	Populus_trichocarpa4A
		Potri.013G022900.2	GUX 4	Populus_trichocarpa4B
*Saccharum spp* (Vettore *et al.*, 2003)	5	sugarcane_contig1	GUX 1	Saccharum_sp1
		sugarcane_contig2	GUX 2	Saccharum_sp2
		sugarcane_contig3	GUX 3	Saccharum_sp3
		sugarcane_contigXA	GUX X	Saccharum_spXA
		sugarcane_contigXB	GUX X	Saccharum_spXB
*Setaria italica†* (v2.2)	5	Seita.5G402400.1	GUX 1	Setaria_italica1
		Seita.9G515500.1	GUX 2	Setaria_italica2
		Seita.3G235400.1	GUX 3	Setaria_italica3
		Seita.1G193600.1	GUX X	Setaria_italicaXA
		Seita.5G386200.1	GUX X	Setaria_italicaXB
*Solanum tuberosum†* (v4.03)	5	PGSC0003DMT400020680	GUX 2	Solanum_tuberosum2A
		PGSC0003DMT400020678	GUX 2	Solanum_tuberosum2B
		PGSC0003DMT400063796	GUX 3	Solanum_tuberosum3
		PGSC0003DMT400048884	GUX 4	Solanum_tuberosum4A
		PGSC0003DMT400048888	GUX 4	Solanum_tuberosum4B
*Sorghum bicolor†* (v3.1)	5	Sobic.003G376700.1	GUX 1	Sorghum_bicolor1
		Sobic.001G479800.1	GUX 2	Sorghum_bicolor2
		Sobic.009G144200.1	GUX 3	Sorghum_bicolor3
		Sobic.004G177000.1	GUX X	Sorghum_bicolorXA
		Sobic.003G360500.1	GUX X	Sorghum_bicolorXB
*Theobroma cacao†* (v1.1)	5	Thecc1EG001429t2	GUX 1	Theobroma_cacao1
		Thecc1EG033846t1	GUX 2	Theobroma_cacao2
		Thecc1EG035450t1	GUX 3	Theobroma_cacao3
		Thecc1EG026564t1	GUX 4	Theobroma_cacao4A
		Thecc1EG026565t1	GUX 4	Theobroma_cacao4B
*Vitis vinifera†* (Genoscope.12x)	3	GSVIVT01026525001	GUX 1	Vitis_vinifera1
		GSVIVT01009501001	GUX 2	Vitis_vinifera2
		GSVIVT01000046001	GUX 4	Vitis_vinifera4
*Zea mays†* (Ensembl-18)	7	GRMZM2G365544_T01	GUX 1	Zea_mays1A
		GRMZM2G135743_T02	GUX 1	Zea_mays1B
		GRMZM2G002023_T02	GUX 1	Zea_mays1C
		GRMZM2G109431_T01	GUX 2	Zea_mays2
		GRMZM2G058472_T02	GUX 3	Zea_mays3
		GRMZM2G031581_T01	GUX X	Zea_maysXA
		GRMZM2G441987_T01	GUX X	Zea_maysXB

* Accessions retrieved from TAIR database (https://www.arabidopsis.org/)

† Accessions retrieved from Phytozome v12 database (https://phytozome.jgi.doe.gov/)

° Accessions retrieved from SUCEST database (http://sucest-fun.org/); ESTs from sugarcane contigs are available in Table S1.

Since the five GUX protein sequences for *Arabidopsis thaliana* were already characterized by Mortimer *et al.* (2010) and Rennie *et al.* (2012), we retrieved their sequences from GenBank. For the other 17 species described above (except for sugarcane), we developed a workflow to standardize the identification of GUX proteins based on gene search and protein domain/motif analyses described by Kumar *et al.* (2016) (Figure S1). For this purpose, we retrieved all protein sequences (only from primary transcripts) from the latest version of their reference genome available in Phytozome v12. All GUX enzymes have the glycosyl transferase family 8 (GT8) domain, which is responsible for the addition of glucuronosyl substitutions onto the xylan backbone (Rennie *et al.*, 2012). Therefore, we screened all protein sequences with a hidden Markov model (HMM) search (*hmmsearch* from HMMER v3.1b2) using the GT8 HMM available on PFAM (PF01501). Since not all proteins that have the GT8 domain are GUX proteins, we sought to identify a GUX specific motif. For this purpose, we performed MEME analysis (Bailey *et al.*, 2009) using the five GUX protein sequences described for *Arabidopsis* (Mortimer *et al.*, 2010; Rennie *et al.*, 2012) and two sequences of rice identified by HomoloGene (Database Resources of the National Center for Biotechnology Information, 2016) as input. The motif present in all those GUX sequences was used to screen all GT8 protein sequences in a subsequent HMMER analysis (Figures S2 and S3). Finally, we defined putative GUX sequences for each species when both GT8 domain and the GUX specific motif were present.[Bibr B39]

Among the 18 species surveyed, sugarcane is the only one that does not have a reference genome available in Phytozome. Thus, we identified its GUX proteins by performing BLAST searches in the SUCEST database (Vettore *et al.*, 2003) using the sorghum orthologs as queries. Then, we used the CAP3 contig assembly program (Huang and Madan, 1999) with the expressed sequence tags (ESTs) obtained from the BLAST search to assemble contigs for each GUX gene in sugarcane. For contigs with incomplete transcripts the closest sorghum ortholog was used to complete the sequence.

After identifying GUX protein sequences for each species, we aligned them with MAFFT (Katoh and Standley, 2013) using the iterative refinement method L-INS-I and no treatment were done in the aligned sequences. Maximum likelihood phylogenetic analysis of the GUX multiple sequence alignment was performed using IQ-Tree v1.6.1 (Trifinopoulos *et al.*, 2016). Branch support was acquired by 1,000 ultrafast bootstraps pseudoreplicates (Minh *et al.*, 2013), under JTT+I+G4 model identified by ModelFinder (Kalyaanamoorthy *et al.*, 2017). For the Bayesian phylogenetic analysis, we used MrBayes v3.2.6 (Ronquist and Huelsenbeck, 2003), using 1,000,000 generations, sample frequency of 500 and diagnostic frequency of 5,000, under JTT+I+G model of evolution. Phylogenetic trees were visualized and edited in FigTree v1.4.3 (Rambaut, 2009). We also tested for robustness of clade arrangements by performing the same analyzes with coding sequences (Figure S4), complete gene sequences (including introns, exons, and UTRs), and gene sequences plus 5’ flanking 1 kb and 3’ flanking 1 kb. In all cases cited above, the results were very similar.

We performed exhaustive HMM searches to identify GUX proteins in several representatives of plant groups, most with economic importance. The number of GUX varied from one to eleven among the species surveyed (Table 1), suggesting a gene family with a complex history of specific-lineages duplications. From 18 plant species, seven of them (*Brachypodium distachyon*, *Setaria italica, Solanum tuberosum*, *Theobroma cacao*, *Arabidopsis thaliana*, *Sorghum bicolor* and *Saccharum* spp.) have five GUX proteins in their genome, whereas five species have more than five orthologs: *Zea mays* and *Brassica oleraceae* have seven GUX, *Brassica rapa* has 10, *Glycine max* has 11, and *Populus trichocarpa* has six. On the other hand, six out of 18 species have less than five GUX: *Eucalyptus grandis* has four GUX proteins, *Oryza sativa*, *Vitis vinifera* and *Citrus sinensis* have three, and both *Marchantya polymorpha* and *Physcomitrella patens* have only one GUX.

Using putative GUX proteins identified *in silico* for each species and their aligned sequences, we reconstructed the phylogenetic trees. Maximum likelihood and Bayesian phylogenies arranged the GUX family into well-supported clades, allowing us to define the orthologous and paralogous relationships (Figure 1). The only exception was for the clade called GUX ‘X’, which is composed of few monocots GUX proteins arranged in different places of the tree depending on the dataset used (nucleotides or aminoacids), and hence we could not establish with complete confidence whether these genes are duplications originated from GUX 4 or GUX 1/3. However, the tree derived from aminoacids alignment (Figure 1) presented a stronger support for a relationship with GUX 1/3 (99.6 from bootstrap and 1.0 of posterior probability) than the tree derived from nucleotides alignment that placed this clade as sister of GUX 2 (less than 50 from bootstrap, and 0.5 of posterior probability). Therefore, with caution, we will consider GUX ‘X’ a specific monocot duplication from GUX 1 or 3 gene.

**Figure 1:**
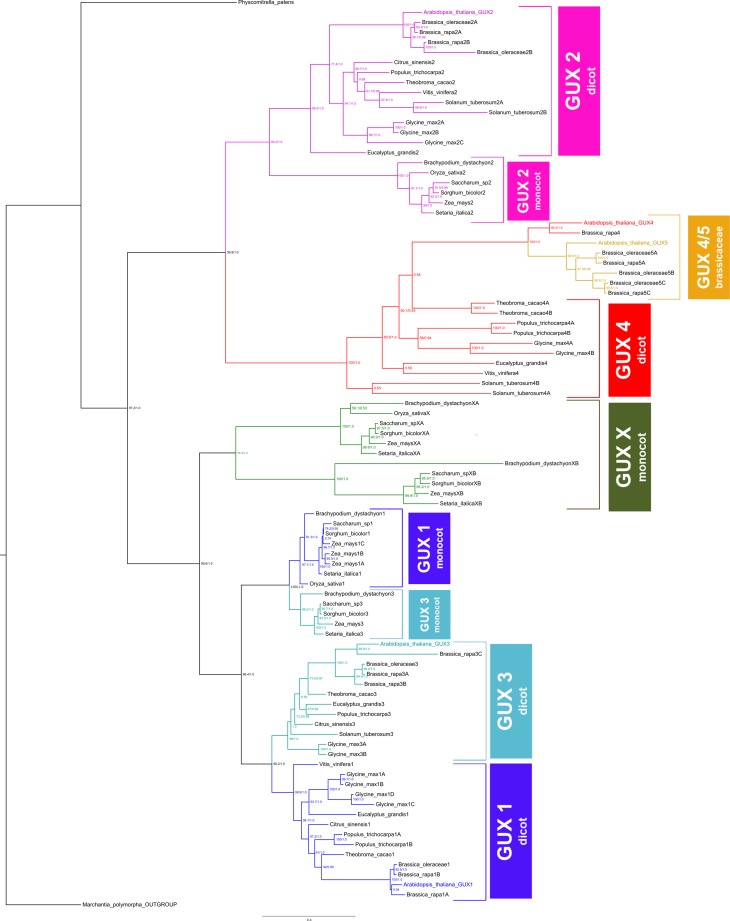
Phylogenetic tree of GUX proteins in plants. Numbers on nodes correspond to the maximum likelihood (ML) ultrafast bootstrap support values followed by Bayesian posterior probabilities. The colored branches are represented by: GUX1 (dark blue), GUX2 (pink), GUX3 (light blue), GUX4 (red), GUX4/5 (yellow) and GUX ‘X’ (green). The GUX sequence from *Marchantya polymorpha* was used as the outgroup.

The GUX proteins are related to the growth and development of cell wall in plants (GUX1 and GUX2 are associated with secondary and GUX3 with primary cell wall development) and have economic importance for biotechnology industry (Lee *et al.*, 2012; Bromley *et al.*, 2013; Mortimer *et al.*, 2015). This highlights the importance of identifying the corresponding genes *in silico* among all species as we showed in our results. With our exhaustive search we were able to identify a great variation among the number of GUX genes in different species. The variation with more than five orthologs may be explained by both ancestral duplications and recent lineage-specific duplications in these plants. For example, at least two late whole-genome duplication events have occurred in *Glycine max* (Schmutz *et al.*, 2010), which can explain the highest number of GUX proteins in this species, with at least two copies of each GUX gene.

It is important to note that we cannot rule out the possibility that some GUX are not included in the genome assembly of these species. Although our description of GUX repertoire suggests a very dynamic evolutionary history, it is still necessary to corroborate these results with improved drafts of some species genomic sequences.

Regarding the evolution pattern observed in the GUX gene family, it can be attributed to a mixture of divergent, concerted and birth-and-death evolutionary models. The divergent model, *i.e.* accumulation of differences between groups that may ultimately lead to the formation of new species/groups (Nei and Rooney, 2005) can be observed in the GUX2 clade. In this case, there is a division between genes from monocots and dicots (Figure 1), indicating that GUX 2 originated before the split between monocots and dicots, and that during evolution they accumulated changes specifics to each group. A similar divergent model was observed in *PHO1* genes, which are involved in phosphate absorption in plants, and where Class II genes from monocots and dicots are separated (He *et al.*, 2013).

The concerted evolution, *i.e.*, members of a gene family evolving in a concerted manner instead of independently (Nei and Rooney, 2005), can be observed in the relationship between the GUX1 and GUX3 clades (Figure 1). Regarding these genes, the phylogenetic tree recovered paralogous clades instead of orthologous clades, indicating that paralogous genes (*e.g.* GUX1 and GUX3 of monocots) are more similar to one another than they are to their true orthologs in closely related species (*e.g.* GUX1 of both monocots and dicots). The clade GUX1 monocot was named this way because BLAST analyses of most of its sequences show the *Arabidopsis* GUX1 as top hits. The same reasoning applies for the clade GUX3 monocot, where most sequences are more similar to *Arabidopsis* GUX3 than GUX1. However, further functional analyses of these proteins are necessary to corroborate the paralog relationship of GUX1 and GUX3 in monocots. The concerted evolution model has also been observed among rice genes from chromosome 11 and 12 that went through a series of genomic modification events until they became more similar among their paralogs than their orthologs (Wang *et al.*, 2007). Furthermore, our analysis of the GUX family revealed characteristics consistent with the birth-and-death evolution model, *i.e.* new genes are originated by successive gene duplication, while some are deleted and others are maintained throughout evolution (Nei *et al.*, 1997), as we have identified lineage-specific patterns of duplication, deletion, and retention of genes among species (Nei and Hughes, 1992). As a result, some species possess fewer GUX (*e.g. Oryza sativa* has lost GUX3 gene), possibly due to deletion or loss-of-function mutations (Figure 2), whereas others possess specific paralogous duplications (*e.g. Solanum tuberosum* has two copies of GUX2 gene, and *Zea mays* has three copies of GUX1 gene). At the same time, we observe that GUX5 is exclusive to the Brassicaceae clade (highlighted in dark red in Figure 2), probably due to a recent duplication of GUX4 specific to this family. According to Blanc *et al.* (2003), *Arabidopsis* experienced two whole genome duplications during its evolution, with the earliest event occurring before the divergence of *Arabidopsis* and *Brassica rapa* (approximately 24-40 Mya). This event may explain the exclusivity of GUX5 in the Brassicaceae family (Figure 2). Accordingly, this Brassicaceae-specific clade was named GUX4/5. Moreover, the uncertainty regarding the GUX ‘X’ placement may indicate that those proteins arose independently from a monocot-specific duplication, and only functional studies will confirm if they belong to one of the five known GUX clades or if they indeed represent a novel GUX group.

**Figure 2:**
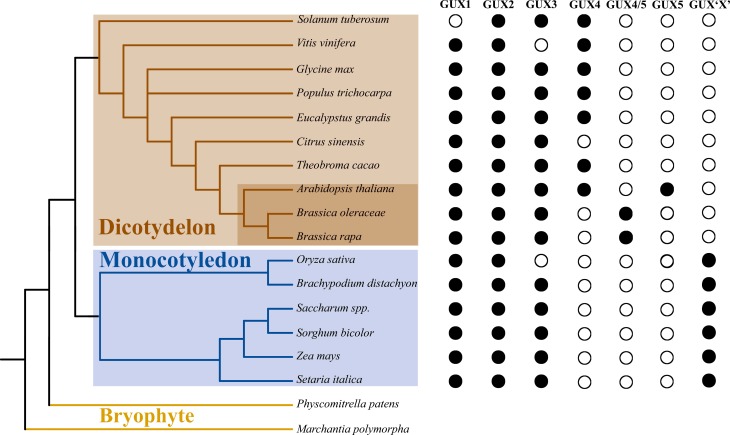
Cladogram representing the relationship among all the species surveyed in this study. Black circles represent the presence of gene(s) within a GUX clade and white circles represent the absence of genes within a clade. The distances do not correspond to phylogenetic distances. Orange box highlights dicotyledonous clade. Dark orange box highlights Brassicaceae family. Blue box highlights the monocotyledonous clade. Yellow lines highlight bryophytes as the outgroup.

Taken together, our results point to a history of ancestral and recent duplications. It is likely that a duplication event has occurred on a common ancestral of dicots and monocots, originating two copies: one that would give rise to GUX2 and one that would undergo another duplication event originating GUX1 and 3. These three genes seem to correspond to the gene set inherited from the common ancestral of monocots and dicots. After the split event around 140–150 Myr ago that gave rise to each group (Chaw *et al.*, 2004), GUX2 duplicated again only in dicots, originating GUX4, which later duplicated one more time only on the Brassicaceae clade, giving rise to GUX5, specific to this family. Monocots, on the other hand, maintained the ancestral set of GUXs 1, 2 and 3, and they are also likely to have a specific ancestral duplication from GUX1 or 3, named here as GUX ‘X’ as explained earlier. The functional differences of GUX 1, 2 and 3 shown in *Arabidopsis* by Bromley *et al.* (2013) and Mortimer *et al.* (2015) provide additional support to the evolutionary divergence demonstrated in this study. Figure S5 depicts this history inferred from our phylogenetic analyses.

Polyploidization followed by diploidization events have been frequent during the evolution of flowering plants, which often led to unpredictable and unexplained genomic variation. Consequently, gene loss, widespread modification of methylation patterns, and nonreciprocal chromosomal exchanges may have happened (Doyle *et al.*, 2008). This could explain part of the differences in the numbers of genes between the plants surveyed and also the dynamic history of this gene family, which shows a mixture of evolutionary models.

The first step towards understanding gene function is to know its evolutionary history in the group of interest. Knowing whether a gene is present in the genome as single or several copies, whether there were specific-lineage losses and gains, or whether the duplicates had evolved with an accelerated rate, can bring important inentendisights to better define the scope of further experimental studies. Our results provide a comprehensive overview of GUX proteins among land plants and also important information on their molecular evolutionary history, showing that this gene family has experienced a mixture of evolution models. This study serves as basis for future genetic engineering studies with the GUX family that aims to increase the efficiency of biofuels production.

## Supplementary material

The following online material is available for this article:

Figure S1 Scheme of the methodology used for the screening of genes.

Figure S2 Conserved motif identified among the five GUX sequences described in *Arabidopsis* and two rice GUX sequences.

Figure S3 GUX motif highlighted in the sequence alignment used to generate the phylogenetic tree.

Figure S4 Phylogenetic tree of *GUX* coding sequences in plants.

Figure S5 A hypothesis of the evolutionary history of GUX genes.

Table S1 ESTs used to produce contigs for each GUX gene in sugarcane.
