# Supplementary material for: Bringing to light the molecular evolution of GUX genes in plants
Source: Genet Mol Biol. 2020 Mar 23;43(1):e20180208. doi: 10.1590/1678-4685-GMB-2018-0208 (PMC7198009; doi:10.1590/1678-4685-GMB-2018-0208)
Supplement: Supplementary file 1 [file 1415-4757-GMB-43-1-e20180208-suppl6.pdf]

Supplementary Material to "Bringing to light the molecular evolution of *GUX* genes in plants"

Table S1 - ESTs from Vettore *et al.* (2003) used to produce contigs for each GUX gene in sugarcane and the contig's respective gene identifiers in the sugarcane monoploid genome (Garsmeur *et al*., 2018; http://sugarcane-genome.cirad.fr/; blastp with e-value = 0).

Acession number	ESTs producing significant alignments (Vettore *et al*., 2003)	Best hits on sugarcane monoploid genome (Garsmeur *et al*., 2018)
sugarcane_contig1	SCCCLR1078C05.g	Sh_251G01_p000110<br>Sh_011C11_p000080<br>Sh_248J12_p000060<br>Sh_249M20_p000010
sugarcane_contig2	SCBFAD1091C02.g<br>SCSGFL4C05H07.g<br>SCCCRZ2003G11.g<br>SCBGFL4055H08.g<br>SCCCFL4119C02.g<br>SCJLFL4099B06.g	Sh_143I09_contig-1_p000030
sugarcane_contig3	SCBFRZ2017F10.g<br>SCJLSB1067D04.g<br>SCQGST1034D01.g<br>SCBFRZ2017F10.g<br>SCAGFL3022E11.g<br>SCQGLB2045H07.g	Sh_011C11_p000080<br>Sh_248J12_p000060<br>Sh_251G01_p000110<br>Sh_249M20_p000010
sugarcane_contigXA	SCRURT3065E10.g<br>SCRURT3065E10.b	Sh_251G01_p000110<br>Sh_248J12_p000060<br>Sh_011C11_p000080
sugarcane_contigXB	SCJLFL4099B06.g<br>SCEQSB1C01A06.g<br>SCCCST3006B11.g<br>SCSBST3102D05.g<br>SCCCST3C06B11.g<br>SCSGSB1006A08.g<br>SCEZST3149H12.g	Sh_210K15_p000010
