# Supplementary material for: Bringing to light the molecular evolution of GUX genes in plants
Source: Genet Mol Biol. 2020 Mar 23;43(1):e20180208. doi: 10.1590/1678-4685-GMB-2018-0208 (PMC7198009; doi:10.1590/1678-4685-GMB-2018-0208)
Supplement: Supplementary file 3 [file 1415-4757-GMB-43-1-e20180208-suppl2.pdf]

Supplementary Material to "Bringing to light the molecular evolution of *GUX* genes in plants"

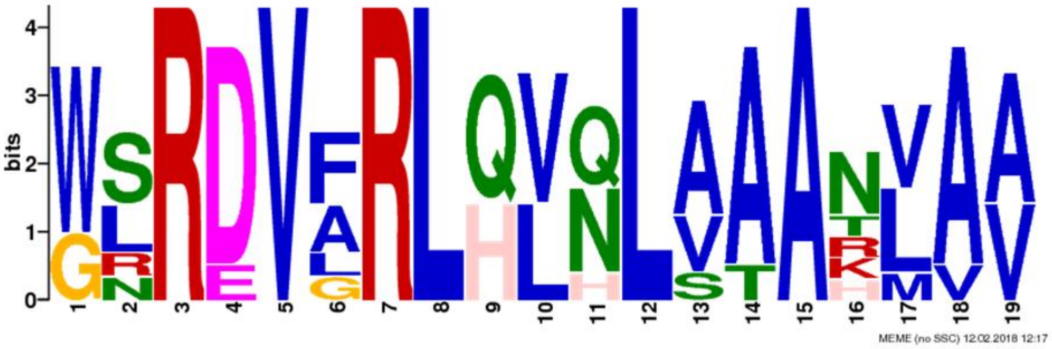

Figure S2 - Conserved motif identified among the five GUX sequences described in *Arabidopsis* and two rice GUX sequences.
