# Supplementary material for: Bringing to light the molecular evolution of GUX genes in plants
Source: Genet Mol Biol. 2020 Mar 23;43(1):e20180208. doi: 10.1590/1678-4685-GMB-2018-0208 (PMC7198009; doi:10.1590/1678-4685-GMB-2018-0208)
Supplement: Supplementary file 5 [file 1415-4757-GMB-43-1-e20180208-suppl4.pdf]

Supplementary Material to "Bringing to light the molecular evolution of *GUX* genes in plants"

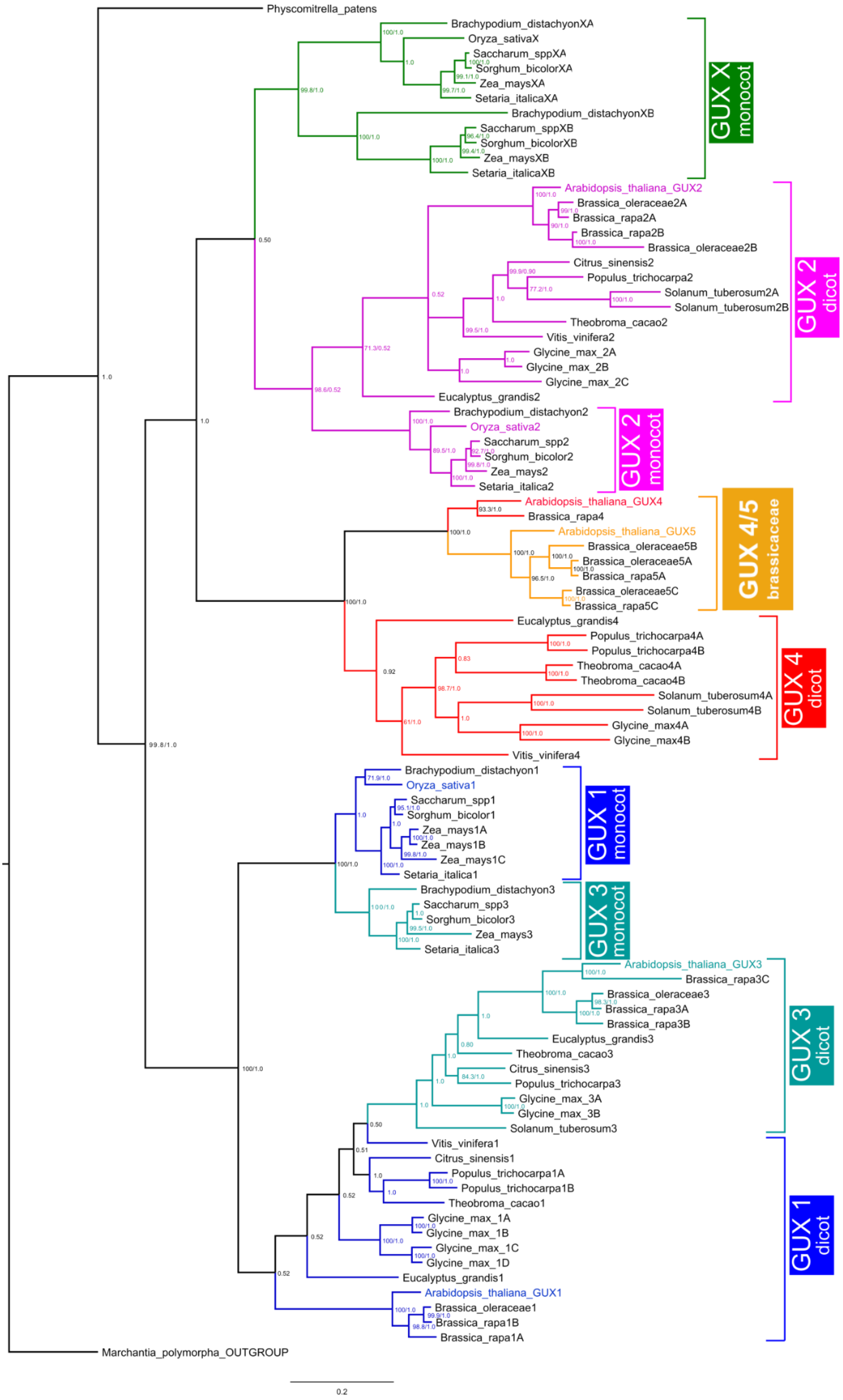

Figure S4 - Phylogenetic tree of *GUX* coding sequences in plants. Numbers on nodes correspond to the maximum likelihood (ML) ultrafast bootstrap support values followed by Bayesian posterior probabilities. The colored branches are represented by: GUX1 (dark blue), GUX2 (pink), GUX3 (light blue), GUX4 (red), GUX4/5 (yellow) and GUX 'X' (green). The GUX sequence from *Marchantya polymorpha* was used as the outgroup.
