# Supplementary material for: Bringing to light the molecular evolution of GUX genes in plants
Source: Genet Mol Biol. 2020 Mar 23;43(1):e20180208. doi: 10.1590/1678-4685-GMB-2018-0208 (PMC7198009; doi:10.1590/1678-4685-GMB-2018-0208)
Supplement: Supplementary file 6 [file 1415-4757-GMB-43-1-e20180208-suppl5.pdf]

Supplementary Material to "Bringing to light the molecular evolution of *GUX* genes in plants"

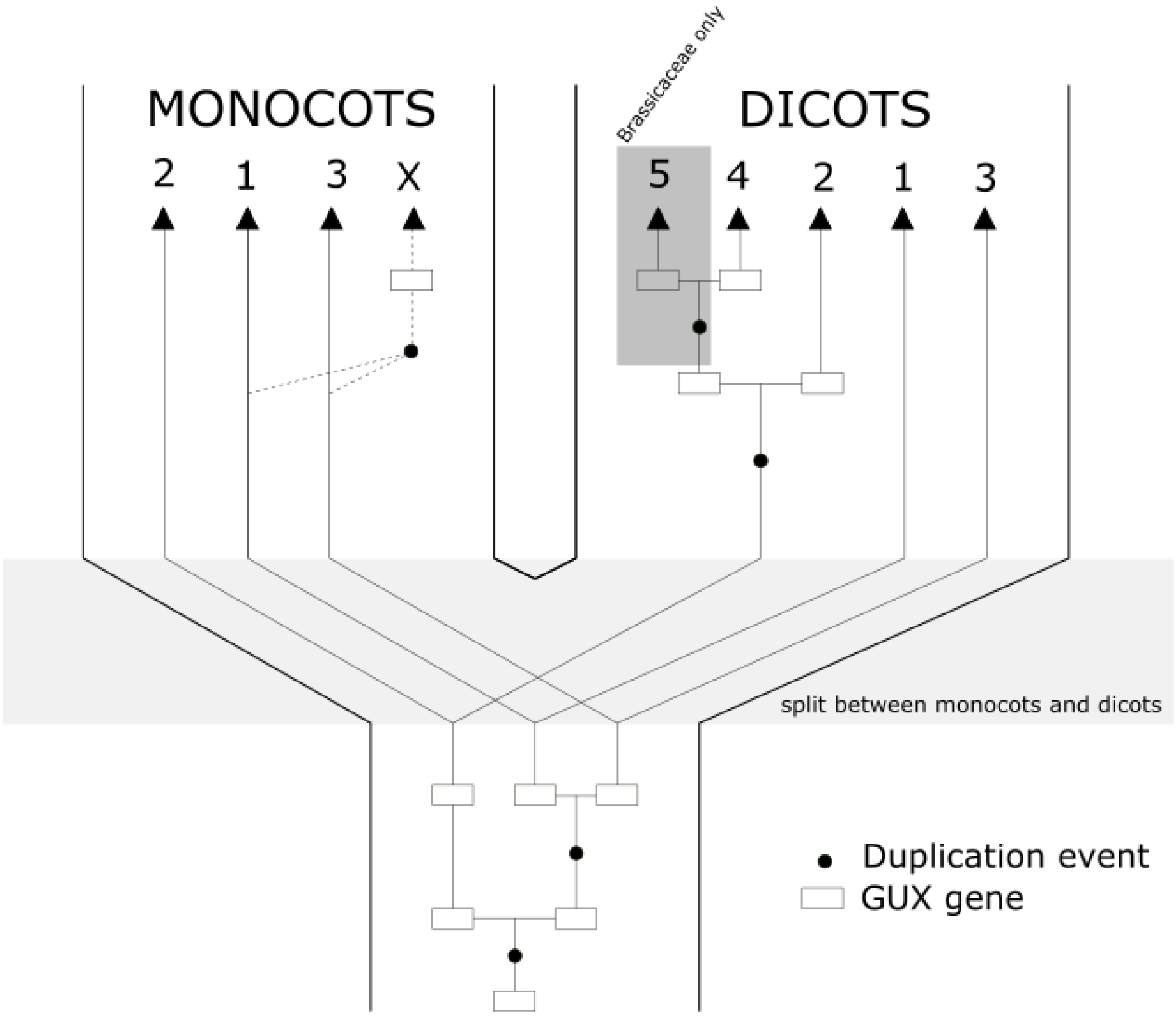

Figure S5 - A hypothesis of the evolutionary history of GUX genes. According to this scheme, the GUX2 gene was originated from an event of duplication on a GUX ancestral gene, while GUX1 and GUX3 were originated from the other copy originated from this duplication event, which occurred before the split between monocots and dicots. In monocots, the GUX'X' was originated after the divergence between monocots and dicots. Likewise, in dicots, GUX4 was the result of a duplication of GUX2. GUX 4 still has undergone a new duplication, originating the clade of GUX5 exclusive of Brassicaceae family. All three genes originated prior to the event of divergence between monocots and dicots remain until the present day in both groups.
